# Supplementary material for: Recently evolved human-specific methylated regions are enriched in schizophrenia signals
Source: BMC Evol Biol. 2018 May 11;18:63. doi: 10.1186/s12862-018-1177-2 (PMC5946405; doi:10.1186/s12862-018-1177-2)
Supplement: Supplementary file 1 — Additional Method, Figures and Tables (DOCX 475 kb) [file 12862_2018_1177_MOESM1_ESM.docx]

***Additional File 1***

**Recently evolved human-specific methylated regions are enriched in schizophrenia signals**

Banerjee et al

# Contents

**Additional Method 2**

**Additional Figures S1-S5 4**

FIGURE S1 4

FIGURE S2 5

FIGURE S3 6

FIGURE S4 7

FIGURE S5 8

**Additional Tables S1-S5 9**

TABLE S1 9

TABLE S2 10

TABLE S3 10

TABLE S4 11

TABLE S5 11

**Additional References 12**

**Additional Method**

**Enrichment Analysis with INRICH**

To assess enrichment for SNPs with different disease significance thresholds in various gene sets, we generated a range of LD-implicated regions through LD clumping in PLINK for index SNPs with *p*-values from 1x10^-3^ to 1x10^-8^. LD Clumps were formed at r^2^=0.5 with the clump range limited to 250kb. INRICH was run on all the sets of LD intervals using default parameters described by Lee *et al* (2012).

All GENCODE V19 genes were used as the background set. The various gene sets tested included genes within a 100 kb flanking region of DMRs, HARs, PARs and NSS markers similar to the procedure described by Xu *et al*, (2015). For NSS markers, genes were assigned via LD blocks of r^2^≥0.8 since NSS markers are single-base markers unlike the HARs and DMRs that are interval regions. GENCODE v19 gene database (last accessed 5^th^ February 2016) was used to map the genes to DMRs, NSS markers and HARs. In their analysis, Xu *et al* had 893 genes within 100 kb of pHARs, 326 genes within 100 kb of mHARs (regions conserved in all mammals which are accelerated in humans) and 305 genes within 100 kb of PARs. In our study, using GENCODE v19, we had 3700, 1316 and 1268 genes within 100 kb of pHARs, mHARs and PARs respectively.

INRICH merges overlapping genes and overlapping LD-implicated intervals to prevent potentially inflated results due to multi-counting of the same genes/intervals. A total of 2510, 1015, 445, 207, 108 and 68 LD-implicated intervals were analyzed respectively for SNPs with *p*-values from 1x10^-3^ to 1x10^-8^ in the schizophrenia GWAS. Similarly, a total of 4321, 2498, 1596, 1130, 892 and 704 intervals were analyzed for the height GWAS. INRICH employs a two-stage procedure for assigning the statistical significance of enrichment of a given interval set with a given gene set. The first stage employs 10,000 permutations to empirically derive the null distribution of the overlap of intervals with gene sets. The second stage employs multiple testing correction via 5000 rounds of bootstrapping. Enrichment of a given interval is then determined by the likelihood of a chance overlap with a gene set over the empirically observed distribution. The final output from INRICH lists gene sets with an empirical *p-*value at a default of *P* = 0.1. This is not the threshold at which INRICH performs the statistical tests. It is only the threshold to control which gene sets are displayed in the output. The corrected *p-*value obtained via bootstrapping is also displayed alongside. Since bootstrapping is a very robust procedure and causes many gene sets that would otherwise be significant at an empirical level to lose significance, an empirical *p-*value default of 0.1 adjusts what gene sets are visible at the end of the analyses. INRICH also outputs global enrichment of unique genes in gene sets at three thresholds that describes an excess of enriched genes at nominal gene-set *P =* 0.001, 0.01 and 0.05 (Lee *et al* (2012)).

## Additional Figures


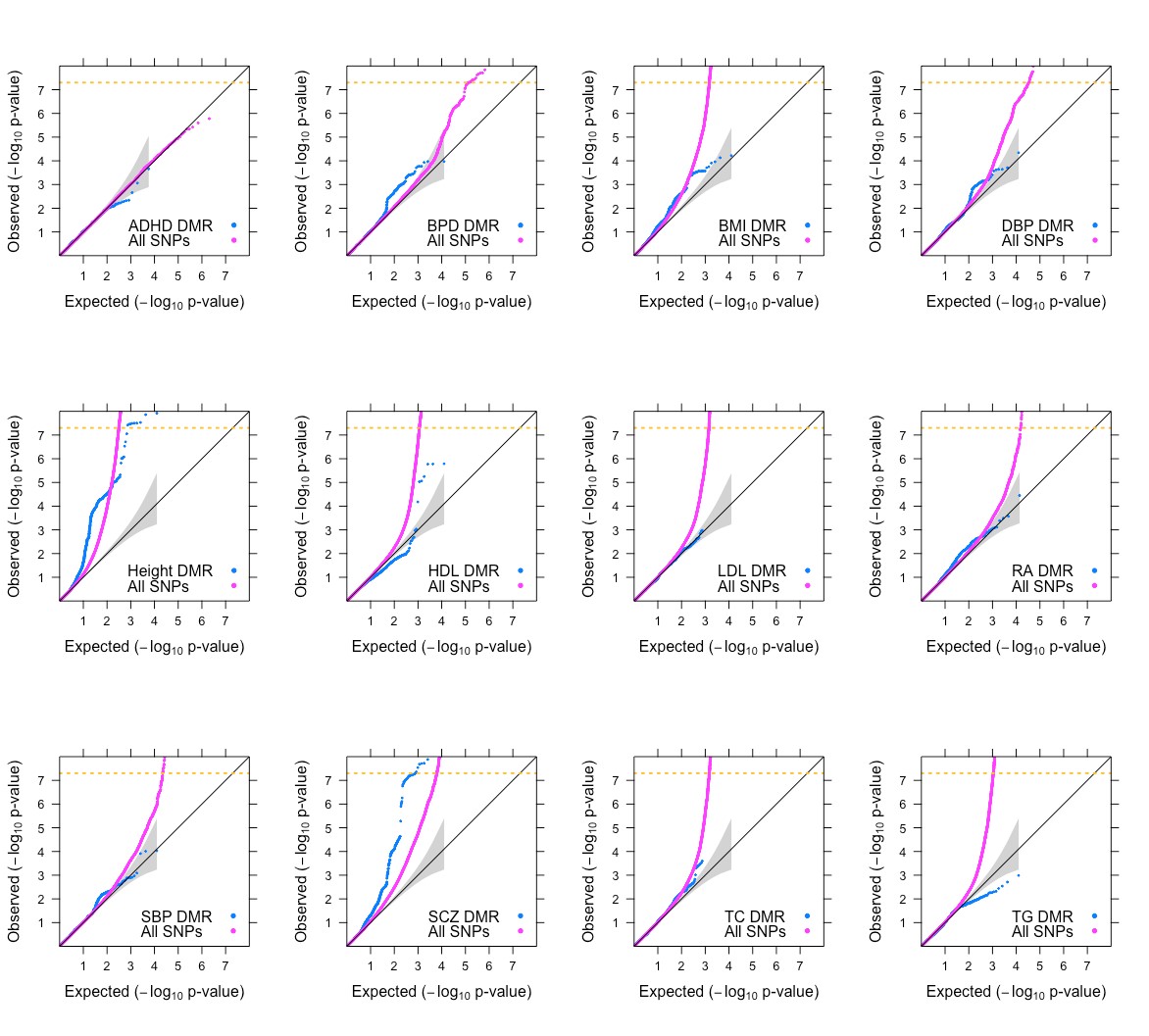


## Figure S1: Enrichment plots for common SNPs shared by all 12 GWAS

Different numbers of SNPs were genotyped in the different GWAS, which could potentially bias our results. To test this, we generated a common set of ~2.4 million SNPs that was determined by intersecting the SNP lists across all twelve GWAS including all SNPs from the ADHD GWAS (~1.2 million SNPs). ADHD, attention deficit hyperactivity disorder; BPD, bipolar disorder; BMI, body mass index; DBP, diastolic blood pressure; HDL, high density lipoprotein; LDL, low density lipoprotein; RA, rheumatoid arthritis; SBP, systolic blood pressure; SCZ, schizophrenia; TC, total cholesterol; TG, triglycerides. We find that the number of SNPs does not influence our results for SCZ, as maximum enrichment is seen for SCZ even when the common set of SNPs is used. The common set of SNPs did not include MHC SNPs, so the MHC was not represented in this analysis.


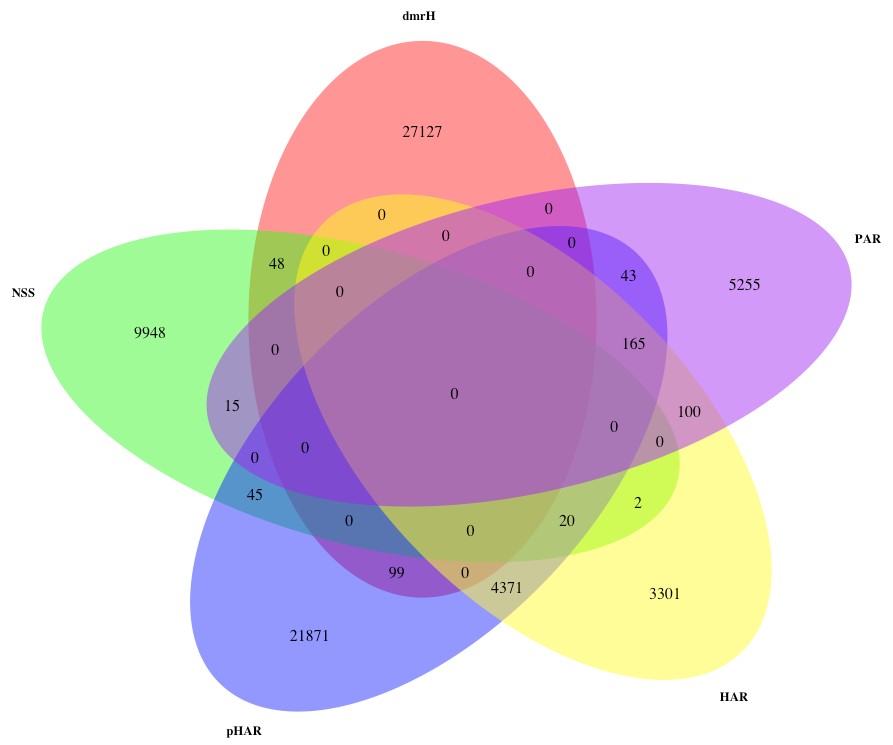


## Figure S2: Overlap of SNPs between various evolutionary annotations

The figure shows that the overlap between the SNPs analysed for enrichment in various evolutionary annotations is very small. SNPs depicted here are those in LD with the respective regions at r^2^ ≥ 0.8. The biggest overlap is between the SNPs in the areas demarcated pHAR (regions conserved in primates that are accelerated in humans) and HAR (regions conserved in mammals that are accelerated in humans), where >50% of SNPs in LD with HARs are also in LD with pHARs. dmrH, human DMRs; NSS, NSS markers; PAR: regions conserved in mammals that are accelerated in primates.


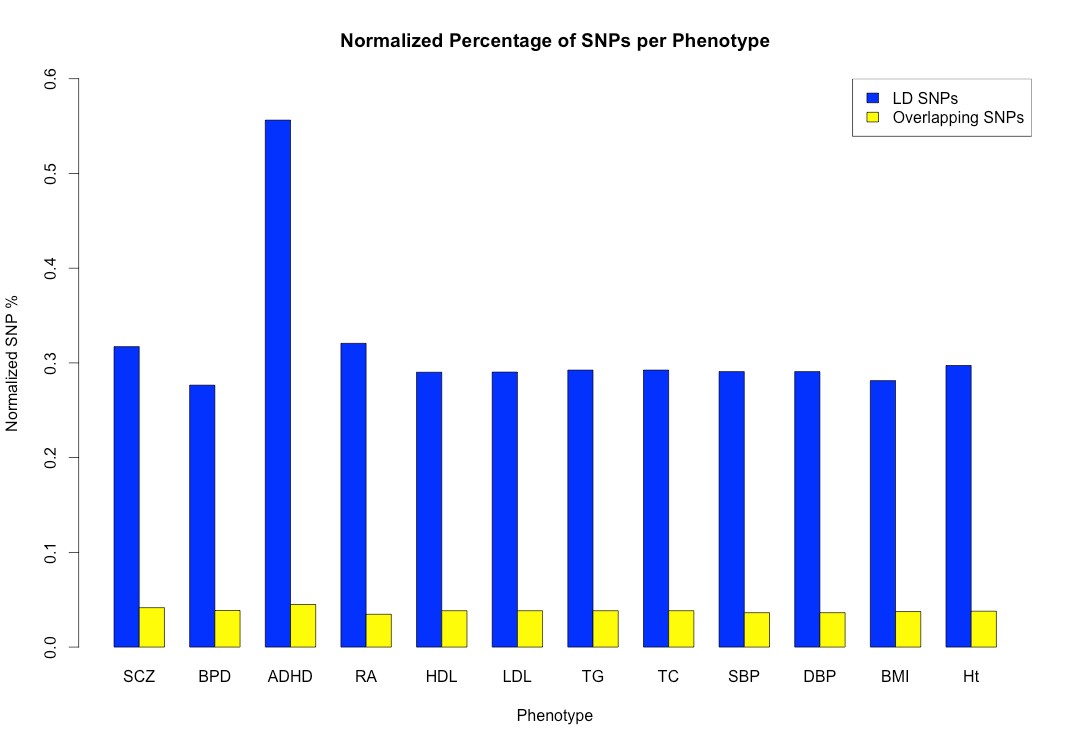


## Figure S3: Proportion of DMR SNPs per GWAS

The figure depicts the normalization performed to determine if the varying number of markers genotyped in different GWAS influences the number of SNPs in DMRs. The total number of SNPs obtained for DMRs in each trait was divided by the total number of SNPs present in the respective GWAS and multiplied by 100 to obtain the normalized percentage of SNPs in DMRs. Blue bars depict the normalized percentage for SNPs in LD with DMRs while yellow bars depict the normalized percentage for SNPs that are physically located within DMRs. We observe that the total number of SNPs genotyped in a GWAS does not influence the proportion of SNPs that are physically within the DMR regions. The same largely holds true for SNPs in LD with DMR regions except for ADHD, possibly because the GWAS was underpowered.


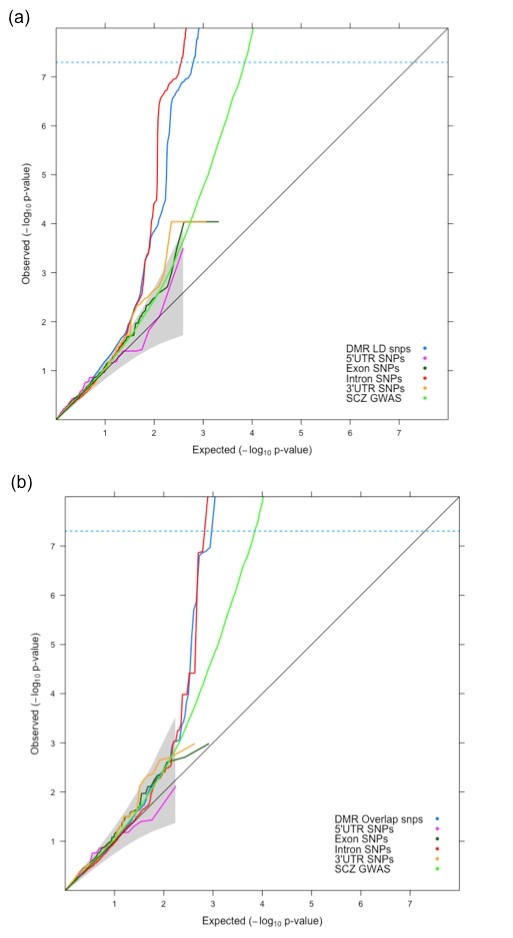


## Figure S4: SCZ SNPs in DMRs stratified by genome annotation

The figure depicts (a) SCZ SNPs in linkage disequilibrium (LD) with DMRs (blue) and (b) SCZ SNPs within DMRs (blue) stratified according to the following genomic annotations: 5’UTR (magenta), Exon (dark green), Intron (red), 3’UTR (orange). The light green line shows all SNPs from the SCZ GWAS.

**
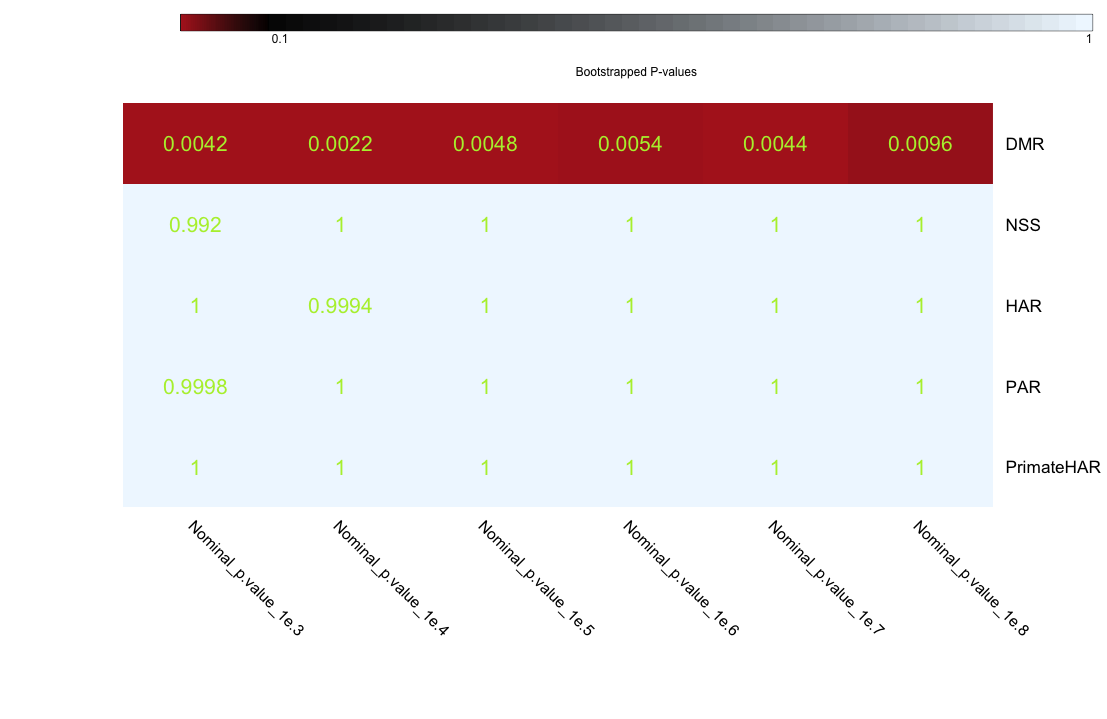
**

**Figure S5: INRICH test for enrichment of association of DMRs, NSS and Accelerated Regions with height**

Corrected *p*-values based on performing multiple testing with bootstrapping 5000 times and *p*=0.1 as threshold. LD clumps of height markers from *p-*value 1e^-3^ – 1e^-8^ were tested for enrichment in: DMR, human-specific DMRs; NSS, Neanderthal Selective Sweep; HAR, mammalian conserved regions that are accelerated in humans; PAR, mammalian conserved regions that are accelerated in primates; and PrimateHAR (pHAR), primate-conserved regions that are accelerated in humans.

## Additional Tables

| **Phenotype** | **Total Study**  **Size (n)** | **Number of SNPs (hg19)** | **Reference** | **SNPs within DMRs** | **SNPs in LD with DMRs** |
| --- | --- | --- | --- | --- | --- |
| Schizophrenia | 150,064 | 9,444,320 | PGC2 (2014) | 3930 | 29,954 |
| Bipolar Disorder | 63,766 | 2,426,991 | Sklar *et al* (2011) | 938 | 6711 |
| Attention Deficit  Hyperactivity  Disorder | 5,415 | 1,206,332 | Neale *et al* (2010) | 542 | 3970 |
| Rheumatoid  Arthritis | 41,282 | 2,553,357 | Stahl *et al* (2010) | 884 | 8187 |
| High Density  Lipoprotein | 99,900 | 2,620,435 | Teslovich *et al* (2010) | 1006 | 7603 |
| Low Density  Lipoprotein | 95,454 | 2,620,568 | Teslovich *et al* (2010) | 1006 | 7608 |
| Triglycerides | 96,598 | 2,620,567 | Teslovich *et al* (2010) | 1006 | 7663 |
| Total Cholesterol | 100,184 | 2,620,450 | Teslovich *et al* (2010) | 1006 | 7663 |
| Systolic Blood  Pressure | 200,000 | 2,461,102 | ICBP GWAS (2011) | 894 | 7157 |
| Diastolic Blood  Pressure | 200,000 | 2,461,102 | ICBP GWAS (2011) | 894 | 7157 |
| Body Mass Index | 339,224 | 2,551,876 | Locke *et al* (2015) | 902 | 7364 |
| Height | 253,288 | 2,545,021 | Wood *et al* (2011) | 964 | 7567 |

## Table S1: Summary of GWAS and DMR SNPs

For each GWAS, the table shows the sample size, marker density, the reference to the specific study, the number of SNPs located within DMRs, and the numbed of SNPs in LD with DMRs.

**Top Canonical Pathways** *P*-value (Fisher’s Exact test)

| Wnt/Ca+ pathway | 1.34E-05 |
| --- | --- |
| P2Y Purigenic Receptor Signalling Pathway | 1.04E-04 |
| Thioredoxin Pathway | 1.07E-04 |
| CREB Signalling in Neurons | 1.45E-04 |
| Synaptic Long Term Potentiation | 1.49E-04 |

**Top Physiological System Development and Function**

| Nervous System Development and Function | 4.41E-02 - 2.23E-02 |
| --- | --- |
| Tissue Morphology | 2.23E-02 - 2.23E-02 |

## Table S2: Pathway analysis results for genes in LD with enriched SNPs in DMRs (Nervous System only).

**Top Canonical Pathways** *P*-value(Fisher’s Exact test)

| Wnt/Ca+ pathway | 1.58E-05 |
| --- | --- |
| P2Y Purigenic Receptor Signaling Pathway | 1.14E-04 |
| CREB Signaling in Neurons | 1.49E-04 |
| Synaptic Long Term Potentiation | 2.05E-04 |
| Thioredoxin Pathway | 2.11E-04 |

**Top Physiological System Development and Function**

| Nervous System Development and Function | 4.42E-02 - 1.41E-02 |
| --- | --- |
| Cardiovascular System Development and Function | 4.42E-02 - 2.24E-02 |
| Connective Tissue Development and Function | 4.42E-02 - 2.24E-02 |
| Hair and Skin Development and Function | 2.24E-02 - 2.24E-02 |
| Hematological System Development and Function | 2.24E-02 - 2.24E-02 |

## Table S3: Pathway analysis results for genes in LD with enriched SNPs in DMRs (All Organ Systems)

**Top Canonical Pathways** *P*-value(Fisher’s Exact test)

| CREB Signalling in Neurons | 1.38E-04 |
| --- | --- |
| IGF-1 Signalling | 6.05E-04 |
| T Cell Receptor Signalling | 1.26E-03 |
| Prolactin Signalling | 1.27E-03 |
| AMPK Signalling | 1.31E-03 |

**Top Physiological System Development & Function**

| Organismal Development | 4.70E-03 - 4.70E-03 |
| --- | --- |
| Tissue Development | 4.70E-03 - 4.70E-03 |
| Nervous System Development and Function | 4.41E-02 - 1.35E-02 |
| Tissue Morphology | 4.41E-02 - 4.41E-02 |

## Table S4: Pathway analysis results for genes in LD with enriched NSS markers (Nervous System only)

**Top Canonical Pathways** *P*-value(Fisher’s Exact test)

| CREB Signalling in Neurons | 1.53E-04 |
| --- | --- |
| Protein Kinase A Signalling | 3.02E-04 |
| Synaptic Long Term Potentiation | 8.90E-04 |
| IGF-1 Signalling | 9.67E-04 |
| ERK/MAPK Signalling | 1.57E-03 |

**Top Physiological System Development & Function**

| Organismal Development | 2.32E-02 - 4.23E-03 |
| --- | --- |
| Tissue Development | 2.32E-02 - 4.23E-03 |
| Nervous System Development and Function | 4.81E-02 - 1.21E-02 |
| Connective Tissue Development and Function | 2.32E-02 - 2.32E-02 |
| Embryonic Development | 2.32E-02 - 2.32E-02 |

## Table S5: Pathway analysis results for genes in LD with enriched NSS markers (All Organ Systems)

**Additional References**

ICBP GWAS: The International Consortium for Blood Pressure Genome-Wide Association Studies. Genetic variants in novel pathways influence blood pressure and cardiovascular disease risk. *Nature* 2011; **478**: 103–109.

Lee PH *et al.* INRICH: interval-based enrichment analysis for genome wide association studies. *Bioinformatics* 2012; **28:** 1797-1799.

Locke AE *et al.* Genetic studies of body mass index yield new insights for obesity biology. *Nature* 2015; **518**: 197–206.

Neale BM *et al.* Meta-analysis of genome-wide association studies of attention-deficit/hyperactivity disorder. *Journal of the American Academy of Child and Adolescent Psychiatry* 2010; **49**: 884–897.

PGC2: Schizophrenia Working Group of the Psychiatric Genomics Consortium. Biological insights from 108 schizophrenia-associated genetic loci. *Nature* 2014; **511**: 421–427.

Sklar P *et al.* Large-scale genome-wide association analysis of bipolar disorder identifies a new susceptibility locus near ODZ4. *Nature Genetics* 2011; **43**: 977.

Stahl EA *et al.* Genome-wide association study meta-analysis identifies seven new rheumatoid arthritis risk loci. *Nature genetics* 2010; **42**: 508–514.

Teslovich TM *et al.* Biological, clinical and population relevance of 95 loci for blood lipids. *Nature* 2010; **466**: 707–713.

Wood AR *et al.* Defining the role of common variation in the genomic and biological architecture of adult human height. *Nature Genetics* 2014; **46**: 1173–1186.

Xu K *et al.* Genomic and network patterns of schizophrenia genetic variation in human evolutionary accelerated regions. *Molecular Biology and Evolution* 2015; **32:** 1148-1160.
